# Evaluation of left ventricular blood flow kinetic energy in patients with acute myocardial infarction by 4D Flow MRI: a preliminary study

**DOI:** 10.1186/s12880-024-01310-8

**Published:** 2024-06-05

**Authors:** Xiqing Niu, Yutong Dun, Guoce Li, Houning Zhang, Bin Zhang, Zhibin Pan, Hao Bian, Liqing Kang, Fenghai Liu

**Affiliations:** 1 Department of Magnetic Resonance Imaging, Hebei Medical University affiliated Cangzhou Central Hospital, Cangzhou, Hebei Province 061000 China; 2 Department of Magnetic Resonance Imaging, Cangzhou Central Hospital, Cangzhou, Hebei Province 061000 China; 3 Department of Magnetic Resonance Imaging, North China University of Science and Technology Affiliated Hospital, Tangshan, 063000 China; 4 Department of Medical Imaging, The First Affiliated Hospital of Hebei North University, Zhang Jiakou, 075000 China

**Keywords:** 4D Flow MRI, Myocardial infarction, Kinetic energy, In-plane kinetic energy, MASS

## Abstract

**Purpose:**

To evaluate the intracavity left ventricular (LV) blood flow kinetic energy (KE) parameters using four-dimensional (4D) flow cardiovascular magnetic resonance (CMR) in patients with acute myocardial infarction (AMI).

**Methods:**

Thirty AMI patients and twenty controls were examined via CMR, which included cine imaging, late gadolinium enhancement (LGE) and global heart 4D flow imaging. The KE parameters were indexed to LV end-diastolic volume (EDV) to obtain average, systolic and diastolic KE as well as the proportion of LV in-plane KE (%). These parameters were compared between the AMI patients and controls and between the two subgroups.

**Results:**

Analysis of the LV blood flow KE parameters at different levels of the LV cavity and in different segments of the same level showed that the basal level had the highest blood flow KE while the apical level had the lowest in the control group. There were no significant differences in diastolic KE, systolic in-plane KE and diastolic in-plane KE between the anterior wall and posterior wall (*p* > 0.05), only the systolic KE had a significant difference between them (*p* < 0.05). Compared with those in the control group, the average (10.7 ± 3.3 µJ/mL vs. 14.7 ± 3.6 µJ/mL, *p* < 0.001), systolic (14.6 ± 5.1 µJ/mL vs. 18.9 ± 3.9 µJ/mL, *p* = 0.003) and diastolic KE (7.9 ± 2.5 µJ/mL vs. 10.6 ± 3.8 µJ/mL, *p* = 0.018) were significantly lower in the AMI group. The average KE in the infarct segment was lower than that in the noninfarct segment in the AMI group (49.5 ± 18.7 µJ/mL vs. 126.3 ± 50.7 µJ/mL, *p* < 0.001), while the proportion of systolic in-plane KE increased significantly (61.8%±11.5 vs. 42.9%±14.4, *p* = 0.001).

**Conclusion:**

The 4D Flow MRI technique can be used to quantitatively evaluate LV regional hemodynamic parameters. There were differences in the KE parameters of LV blood flow at different levels and in different segments of the same level in healthy people. In AMI patients, the average KE of the infarct segment decreased, while the proportion of systolic in-plane KE significantly increased.

## Introduction

After acute myocardial infarction (AMI), the myocardial systolic function of the infarct segment is weakened or lost, and left ventricular (LV) contraction becomes asymmetrical. Asymmetric contraction causes the LV wall to experience uneven blood tension, resulting in complex hemodynamic changes that may lead to adverse remodelling and subsequent left heart failure [1–3]. The effect of AMI on LV hemodynamic has been confirmed, and the quantification of LV hemodynamic is highly valuable for determining the prognosis of patients with myocardial infarction [4–6].

Kinetic energy (KE) represents the energy that blood possess due to its motion and could represent an important hemodynamic parameter to evaluate [7]. The KE of blood flow includes both in-plane and through-plane components. In-plane KE is the sum of all KE values in the horizontal direction of the short axis from the base to the apex of the LV. In contrast, through-plane KE refers to the component of blood flow perpendicular to the short axis of the heart.

Compared with traditional cardiac MRI, 4D Flow MRI can evaluate the blood flow state more comprehensively, so as to help us fully understand the development of the disease. At present, some studies have applied 4D Flow KE parameters to patients with AMI, and found that the in-plane KE of left ventricle increased in the group with decreased ejection fraction [8, 9]. However, a pathological increase in in-plane KE may exert heterogeneous hemodynamic force on the LV wall, which leads to further expansion of the endocardium and increased endothelial dysfunction, and may even be related to the occurrence of complications after myocardial infarction. At present, there are few studies on the kinetic energy of regional blood flow based on 4D Flow MRI. The aim of this study was to analyze the changes in KE parameters of regional blood flow in the cardiac cavity of MI patients and to determine the importance of KE in hemodynamic changes of patients with AMI.

## Materials and methods

### Study population

Thirty patients with AMI and twenty age-/sex-matched healthy controls from Cangzhou Central Hospital were retrospectively evaluated between February 2022 and August 2023. The regional ethics committee approved this study (approval number: 2023-222-02), and the requirement for written informed consent was waived.

The inclusion criteria for patients were as follows: a clinical diagnosis of AMI [10]; percutaneous coronary intervention (PCI) performed within 12 h after the onset of chest pain; CMR imaging completed within one week after treatment; and no contraindications for cardiac MRI. The exclusion criteria for patients were as follows: a previous history of vascular reconstruction surgery (coronary artery bypass grafting or PCI); known cardiomyopathy or valvular heart disease; hemodynamic instability lasting more than 24 h after PCI; poor-quality CMR images; or incomplete imaging data [3].

### CMR examination

All control subjects and patients were scanned in a 3.0T scanner (MR750, GE Healthcare, Signa Discovery) with a 16-channel phased array coil. All the subjects were trained to hold their breath at the end of expiration, and ECG gating and respiratory gating were monitored.

### CMR protocol and image acquisition

The CMR protocol was as follows:

1. The following cines were defined using the survey images: horizontal long-axis, 2-chamber, 4-chamber and the LV volume contiguous short-axis stack.
2. All cines were acquired with a balanced steady-state free precession (bSSFP) procedure. The typical parameters for the bSSFP sequence were as follows: flip angle (FA) 60°, echo time (TE) 1.89 milliseconds, repetition time (TR) 3.74 milliseconds, field of view (FOV) 320–420 mm depending on patient size, slice thickness 8 mm, and 25 phases per cardiac cycle.
3. LGE imaging was performed 15 min after gadolinium-based contrast agent injection in AMI patients only. LGE imaging was performed with a phase sensitive myocardial delayed enhancement (PSMDE) spoiled gradient recalled echo (FSPGR) sequence. The PSMDE sequence details are as follows: TE/TR, 2.46/5.3 msec; FA, 25°.
4. For global heart 4D flow, the field of view (FOV) was planned in the transaxial plane to ensure that the global heart was within the FOV. 4D flow data were acquired with PC VIPR, a 3D radially undersampled, three-directional velocity-encoding technique [11]. Scan parameters captured by 4D Flow MRI as defined in the latest version of the 4D Flow MRI Consensus Statement for 2023 [12]: TE, 2.0 ms; TR, 5.3 ms; FA, 7°; VENC, 150 cm/sec; spatial resolution, 2.5mm^3^; temporal resolution,30ms.

### Image analysis

A GE AW4.7 postprocessing workstation was used to measure cardiac function parameters. The indices of left ventricular volume and cardiac function, including the left ventricular end diastolic volume index (LVEDVi), left ventricular end systolic volume index (LVESVi) and left ventricular ejection fraction (LVEF), which were corrected by body surface area, were obtained.

The parameters of KE were analysed using MASS (version 2021-EXP, Medis Medical Imaging). All the images were outlined by a radiologist with 7 years of experience in CMR diagnosis, using automatic tracking technology combined with manual adjustment to outline the endocardium and epicardium of the left ventricle at end diastole and end systole, and were confirmed or adjusted by a radiologist with more than 10 years of experience. The calculation formula for KE was KE = 1/2 ρ_blood_ × V_voxel_× v^2^, where ρ_blood_ represents the density of blood (1.06 g/cm3), V_voxel_ represents the voxel volume, and v represents the velocity. For each phase, the total KE within the LV was obtained by summing the KE of each voxel, and then the time-resolved KE curve was obtained by summing the KE values of each voxel in the whole cardiac cycle to derive physiologically relevant parameters. In-plane KE was obtained by summing all KE in the short-axis planes from basal to apical LV and is expressed as a percentage of the total LV KE. All KE parameters were indexed to the LVEDV, and the units were µJ/mL (KEi_EDV_) [13].

**Fig. 1:**
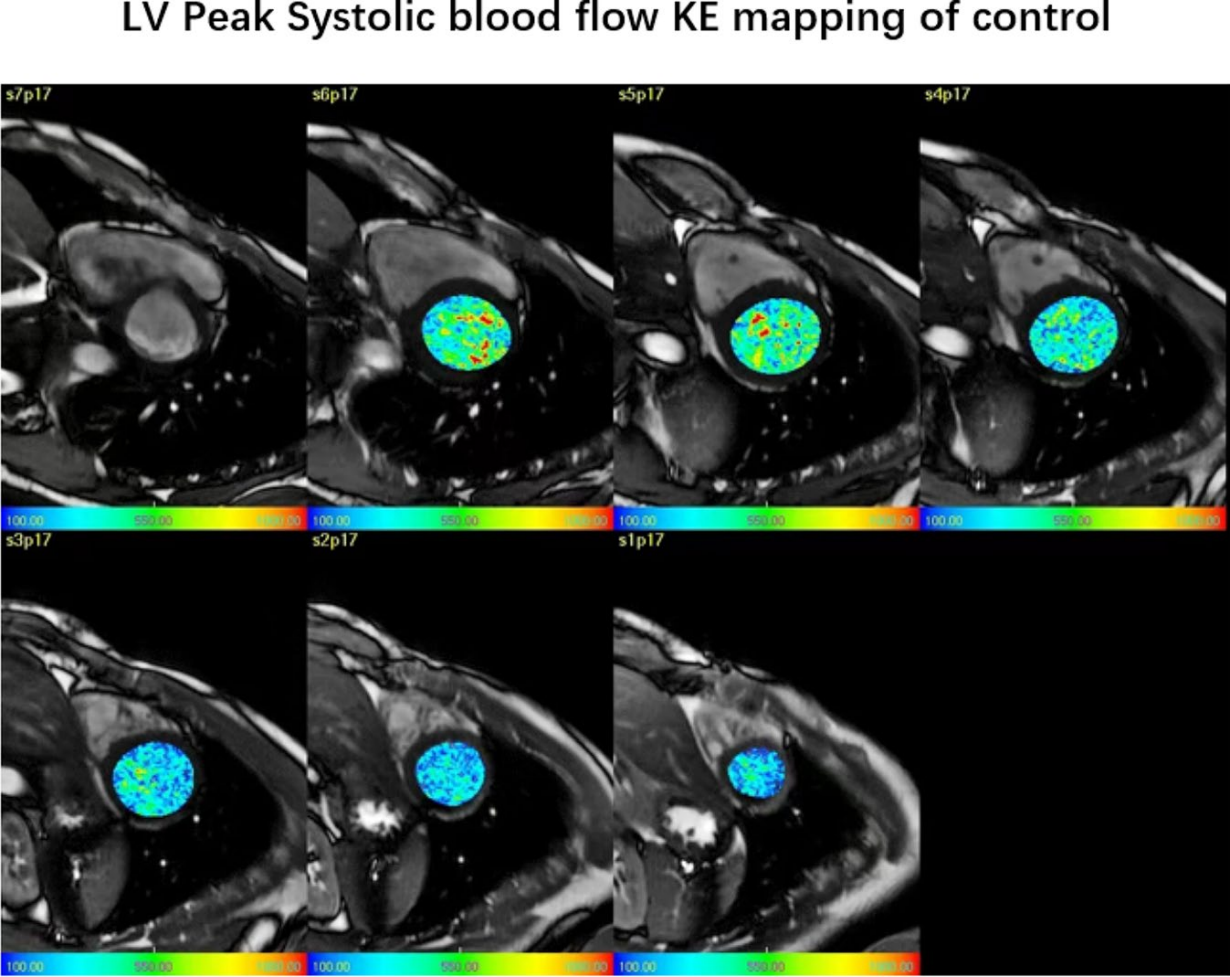
An example of left ventricular short-axis KE mapping in the healthy control group, the parameters of the healthy control group are as follows: LVEF = 67.1%±9.4, Systolic KEi_EDV_=18.9 ± 3.9µJ/ml, In-plane KE = 30.9%±12.2

**Fig. 2:**
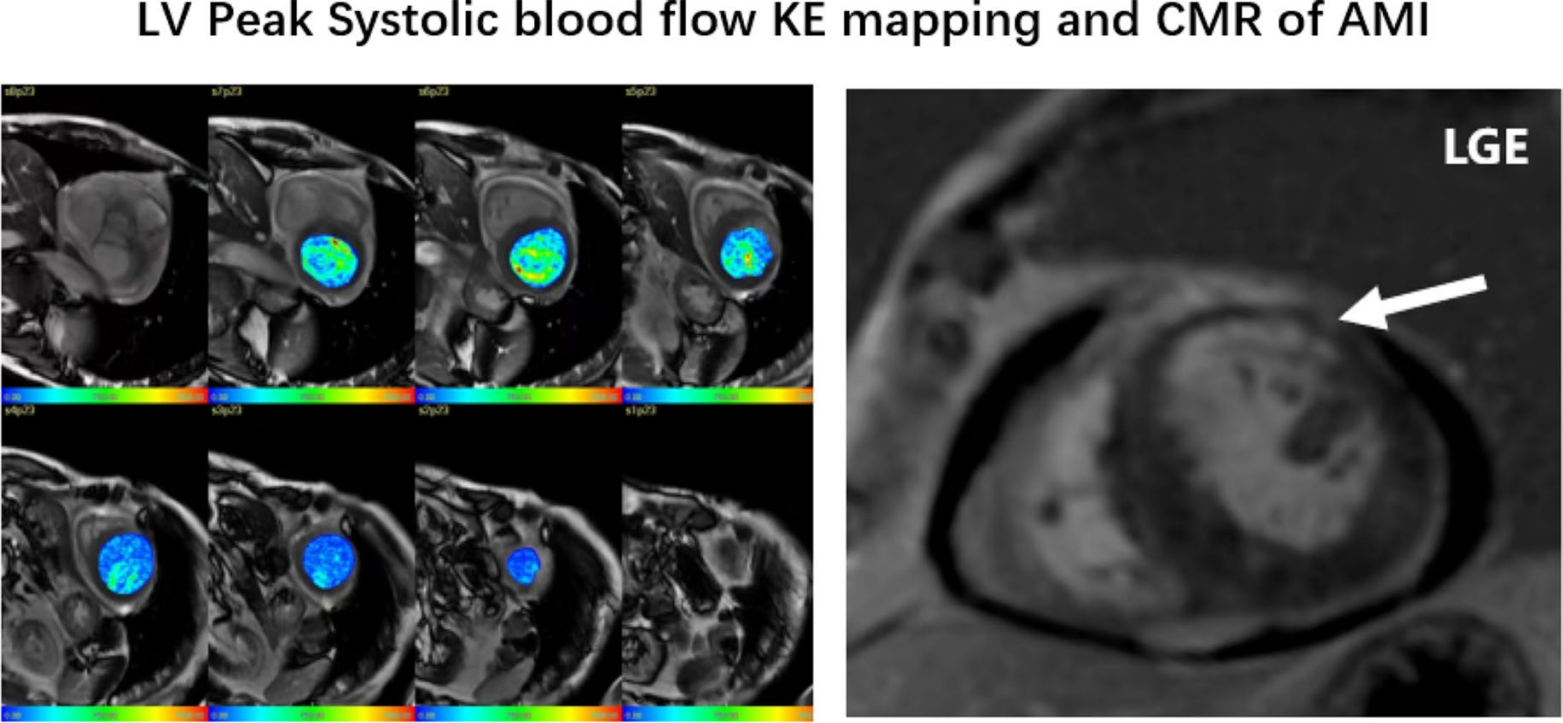
An example of left ventricular short-axis KE mapping and CMR in the AMI group, and the arrows refers to the infarction area in the LGE images of AMI patients in the anterior wall. The parameters of the AMI group are as follows: LVEF = 48.9%±13.0, systolic KEi_EDV_=14.6 ± 5.1µJ/ml, In-plane KE = 32.0%±11.4. LGE: delayed enhancement

### Segmentation method for calculating the regional KE of the LV cavity

MASS automatically quantified the blood flow KE parameters in 16 segments of the heart in all subjects according to American Heart Association standards. In this study, the subjects’ ventricles were divided in equal thirds into base, mid-ventricle and apex, and each level was divided into 4 segments (anterior wall, posterior wall, septal wall and lateral wall). The differences in regional blood flow KE at different levels and in different segments of the same level of the heart cavity in the healthy control group were used as the reference for the AMI group. In the AMI group, the segment with the largest area of myocardial infarction was defined as the infarct segment, and the contralateral myocardium was defined as the noninfarct segment.

### Statistical analysis

All analyses were performed using SPSS® Statistics (Version 26.0, International Business Machines). The Shapiro-wilk test was used to evaluate the normal distribution of the data. The normally distributed data are expressed as the mean ± standard deviation (SD), and two-independent sample t tests were used. Categorical data are presented as numbers and proportions and Chi-square test were used. The comparison of different levels and different segments in the control group was performed by one-way ANOVA, and the difference in regional blood flow KE in the AMI group was compared by paired t test or Wilcoxon signed rank sum test. A two-tailed p value of less than 0.05 was considered to indicate statistical significance.

## Results

### Patient demographic characteristics

There were no significant differences in heart rate (67.1 ± 15.7 beats/min vs. 73.0 ± 8.8 beats/min, *P* = 0.057) between the AMI patients and the control group. Healthy controls and the AMI group were matched for age (54 ± 9 years vs. 58 ± 9 years, *P* = 0.133). The CMR volume parameters of the AMI patients were significantly greater than those of the control group, while the LVEF was significantly lower (48.9%±13.0 vs. 67.1%±9.4) (Table 1).

**Table 1:** Comparison of demographic data between controls and AMI patients

	Controls (*n* = 20)	AMI patients (*n* = 30)	t/χ2	*P*
Sex (male: female) ^a^	11:9	18:12	0.123	0.726
Hypertension (%) ^a^	65	60	0.127	0.721
Age (years) ^b^	53.8 ± 9.8	58.1 ± 9.7	-1.528	0.133
Heart rate (bpm) ^b^	73.0 ± 8.8	67.1 ± 15.7	1.950	0.057
LVEF (%) ^b^	67.1 ± 9.4	48.9 ± 13.0	1.198	< 0.001
LVEDVi (ml/m^2^) ^b^	67.5 ± 17.4	99.8 ± 19.8	-6.487	< 0.001
LVESVi (ml/m^2^)	26.4 ± 8.6	52.3 ± 15.3	0.030	< 0.001

The values were presented as the mean ± standard deviation (SD). ^a^ Values are counted (n) and Chi-square test were used. ^b^ Values are normally distributed data and two-independent sample t tests were used. LV measurements were indexed to body surface area. Abbreviations: LVEF, left ventricular ejection fraction. LVEDVi, left ventricular end-diastolic volume (indexed). LVESVi, left ventricular end-systolic volume (indexed).

### KE parameter results

#### Total KE of LV blood flow for the two groups

The average LV KEi_EDV_ and systolic and diastolic KE in AMI patients were significantly lower than those in the control group (10.7 ± 3 µJ/ml vs. 14.7 ± 3 µJ/ml, *P* < 0.01; 14.6 ± 5.1 µJ/ml vs. 18.9 ± 3.9 µJ/ml, *P* = 0.003; and 7.9 ± 2.5 µJ/ml vs. 10.6 ± 3.8 µJ/ml, *P* = 0.003, respectively). No significant difference was found in the in-plane KE proportion between the AMI patients and the control group (32.0%±11.4 vs. 30.9%±12.2, *P* = 0.742) (Table 2; Figs. 1 and 2).

**Table 2:** Comparison of LV blood flow KE parameters between controls and AMI patients

	Controls (*n* = 20)	AMI patients (*n* = 30)	t	*P*
LV KEi_EDV_ (µJ/ml)	14.7 ± 3.6	10.7 ± 3.3	4.006	< 0.001
Systolic KEi_EDV_ (µJ/ml)	18.9 ± 3.9	14.6 ± 5.1	3.172	0.003
Diastolic KEi_EDV_ (µJ/ml)	10.6 ± 3.8	7.9 ± 2.5	2.973	0.010
In-plane KE (%)	30.9 ± 12.2	32.0 ± 11.4	-0.331	0.742

### Blood flow KE at different levels in the control group

As shown in Table 3, blood flow KE parameters were different at different levels in the same patient. The average, systolic and diastolic KE in the basal segment (6.7 ± 1.8 µJ/ml, 8.5 ± 2.7 µJ/ml, and 5.4 ± 2.1 µJ/ml, respectively) were significantly greater than those in the middle segment (4.9 ± 2.1 µJ/ml, 5.9 ± 2.6 µJ/ml, and 4.5 ± 2.2 µJ/ml, respectively) and apical segment (1.7 ± 0.6 µJ/ml, 2.0 ± 0.7 µJ/ml, and 1.6 ± 0.6 µJ/ml, respectively), and the values in the apical segment were the lowest (all *p* < 0.05).

**Table 3:** Blood flow KE at different levels of the LV cavity in controls

	Base	Mid-ventricle	Apex	F	*P*
Average KEi_EDV_ (µJ/ml)	6.7 ± 1.8	4.9 ± 2.1^**a**^	1.7 ± 0.6^**ab**^	45.799	< 0.001
Systolic KEi_EDV_ (µJ/ml)	8.5 ± 2.7	5.9 ± 2.6^**a**^	2.0 ± 0.7^**ab**^	42.645	< 0.001
Diastolic KEi_EDV_ (µJ/ml)	5.4 ± 2.1	4.5 ± 2.2	1.6 ± 0.6^**ab**^	24.536	< 0.001
In-plane KE (%)	33.6 ± 7.7	32.6 ± 13.5	41.0 ± 18.5	2.159	0.125
Systolic in-plane KE (%)	37.7 ± 8.8	30.2 ± 15.1	34.1 ± 19.0	1.220	0.303
Diastolic in-plane KE (%)	28.8 ± 10.0	35.9 ± 13.5	48.4 ± 18.5^**ab**^	9.122	< 0.001

^a^ Compared with the basal level, *P* < 0.05; ^b^ compared with the middle level, *P* < 0.05; pairwise comparisons were calibrated by Bonferroni multiple correction.

### Blood flow KE in different segments in the control group

**Fig. 3:**
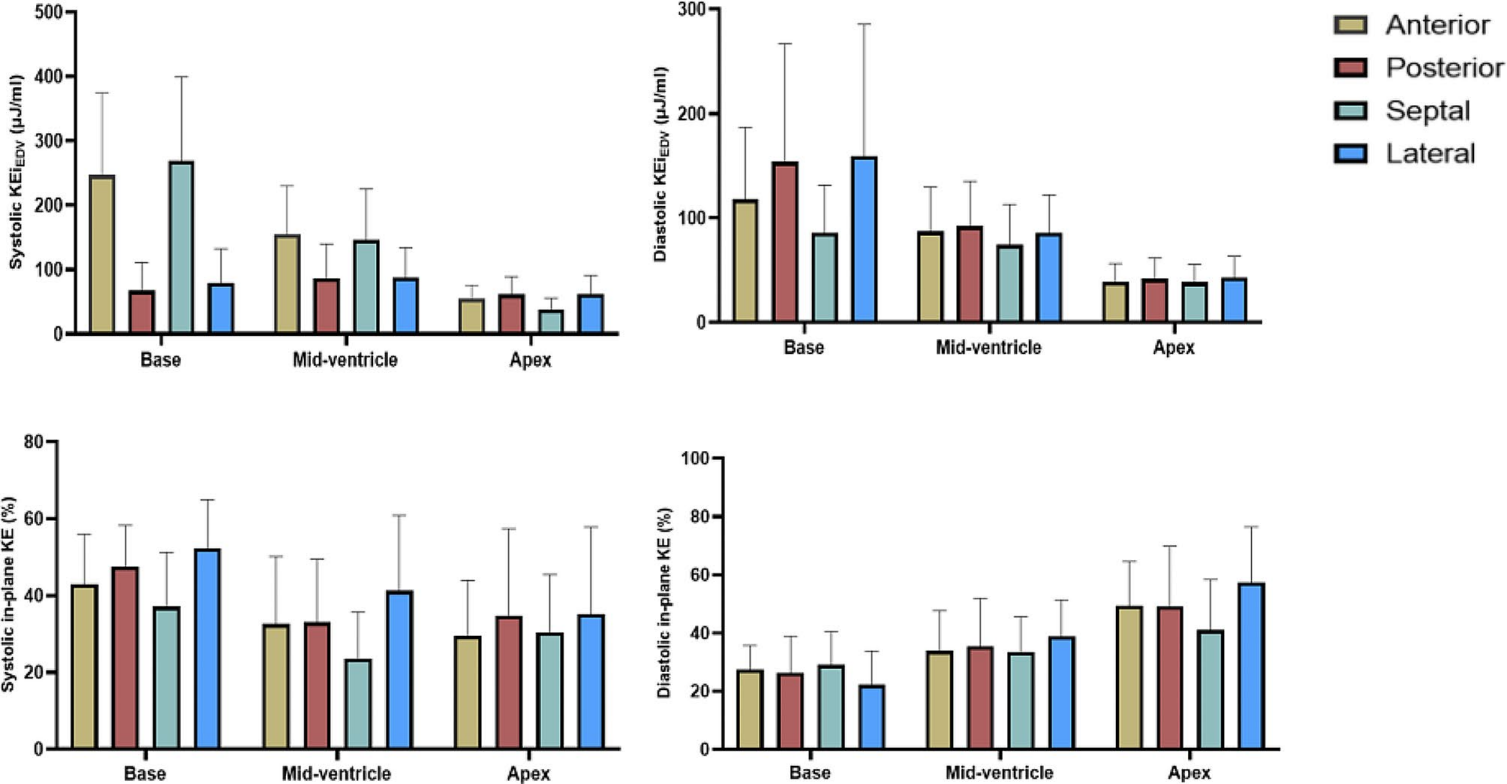
Bar charts of LV flow KEi_EDV_ parameters at different segments of the LV cavity in controls (bars = mean ± sd; error bars = inter-quartile range). Abbreviations are the same as those in Table 4

Comparisons of LV blood flow KE parameters in different segments at the same level in the control group were shown in Table 4; Fig. 3. The systolic KE and systolic in-plane KE were significantly different, while the diastolic KE and diastolic in-plane KE were not significantly different. According to pairwise comparisons of the anterior wall, posterior wall, septal wall and lateral wall, the systolic blood flow KE in the anterior wall and septal wall was significantly greater than that in the contralateral myocardial segment (all *P* < 0.05). The systolic in-plane KE was not significantly different between the anterior and posterior walls but was significantly different between the septal and lateral walls.

**Table 4:** Blood flow KE at different segments of the LV cavity in controls

	Anterior	Posterior	Septal	Lateral	F	*P*
Systolic KEi_EDV_ (µJ/ml)						
Base Mid-ventricle Apex	247.5 ± 127.0^**b**^	67.6 ± 43.8^**ac**^	292.5 ± 203.3^**b**^	78.8 ± 53.2^**acb**^	17.052	**< 0.001**
	154.8 ± 75.5^**b**^	86.6 ± 53.2^**ac**^	147.2 ± 78.4^**b**^	88.6 ± 45.6^**ac**^	6.450	**0.001**
	55.1 ± 20.6	61.8 ± 27.5	54.4 ± 20.4	62.3 ± 28.7	0.599	0.618
Diastolic KEi_EDV_ (µJ/ml)						
Base Mid-ventricle Apex	117.8 ± 69.0	154.0 ± 113.0	85.6 ± 46.0	159.5 ± 126.0	2.681	0.053
	87.4 ± 42.3	92.2 ± 43.0	74.3 ± 38.7	86.1 ± 36.8	0.723	0.542
	39.1 ± 17.2	41.9 ± 19.7	38.4 ± 17.1	43.2 ± 19.9	0.305	0.821
Systolic in-plane KE (%)						
Base Mid-ventricle Apex	42.9 ± 13.0	47.5 ± 10.7^**c**^	37.2 ± 14.0^**b**^	52.2 ± 12.7^**ac**^	5.192	**0.003**
	32.5 ± 17.5	33.0 ± 16.4	23.7 ± 12.0	41.2 ± 19.7^**c**^	3.717	**0.015**
	31.4 ± 19.4	30.7 ± 16.7	30.3 ± 15.1	35.0 ± 22.6	0.269	0.848
Diastolic in-plane KE (%)						
Base Mid-ventricle Apex	27.6 ± 8.3	26.2 ± 12.6	28.9 ± 11.5	22.3 ± 11.6	1.325	0.273
	33.9 ± 13.7	35.4 ± 16.5	33.4 ± 12.2	33.9 ± 12.3	0.664	0.577
	49.5 ± 15.1	49.1 ± 20.6	46.7 ± 18.9	55.4 ± 16.2	0.855	0.468

^a^ Compared with the anterior segment, *P* < 0.05; ^b^ compared with the posterior segment, *P* < 0.05; ^c^ compared with the septal segment, *P* < 0.05; pairwise comparisons were calibrated by Bonferroni multiple correction.

### Regional blood flow KE in the AMI group

In the AMI group, there were 2 cases of anterior wall infarction, 20 cases of posterior wall infarction (14 cases in the basal segment and 6 cases in the middle segment), 5 cases of septal wall infarction, and 3 cases of lateral wall infarction. Because the sample size of patients with anterior and middle posterior wall infarctions was small, this study analysed only the difference of blood flow KE in patients with posterior basal myocardial infarction. As shown in Table 5, the systolic and diastolic KE in the infarct segment were significantly lower than those in the noninfarct segment (49.5 ± 18.7 µJ/ml vs. 126.3 ± 50.7 µJ/ml, *P* < 0.001; 25.7 ± 9.1 µJ/ml vs. 44.5 ± 21.4 µJ/ml, *P* = 0.005). However, the proportion of systolic in-plane KE in the infarct segment was greater than that in the noninfarct segment (61.8% ± 11.5 vs. 42.9% ± 12.1, *P* = 0.001). There was no significant difference in the proportion of diastolic in-plane KE between the infarct and noninfarct segments (53.6% ± 19.0 vs. 43.2% ± 14.4, *P* = 0.203).

**Table 5:** Paired t test results of blood flow KE in the basal segment of posterior wall AMI

	Noninfarct segment	Infarct segment	t	*P*
Systolic KEi_EDV_ (µJ/ml)	126.3 ± 50.7	49.5 ± 18.7	-5.764	*<0.001*
Diastolic KEi_EDV_ (µJ/ml)	44.5 ± 21.4	25.7 ± 9.1	-6.703	*0.005*
Systolic in-plane KE (%)	42.9 ± 12.1	61.8 ± 11.5	4.035	*0.001*
Diastolic in-plane KE (%)	43.2 ± 14.4	53.6 ± 19.0	1.339	0.203

## Discussion

### Total KE of LV blood flow in the control and AMI groups

The KEi_EDV_ parameter of LV blood flow was decreased in patients with AMI, which was consistent with the results of Pankaj et al. [1] and Kanski et al. [14]. After MI, the regional contractility of the myocardium was reduced, which reduces the regional pressure on the blood in the cavity and the pressure gradient between the LV and the aorta, thus reducing the total thrust of systolic blood flow, which ultimately manifests as a decrease in systolic KE. Another finding of the study by Pankaj Garg also supports this view: the LV stroke volume was related to the KEi_EDV_ only at the end of systole in the infarction group but not in the control group. In addition, Riva’s study also found that the KE of patients in the AMI group was significantly reduced [15].

### Blood flow KE at different levels in the control group

Several studies have reported the application of MRI in detecting LV blood flow KE in healthy individuals. However, the current studies on blood flow KE parameters in healthy people have focused mainly on evaluating flow in the global heart chamber, while few studies have evaluated the KE parameters of different segments. In our study of 20 healthy subjects, the KE values at different levels were different: the basal segment had the largest values, while the apex had the smallest values, which may be related to the proximity of the base flow to the left ventricular outflow tract (LVOT). The jet through the aortic valve improved the KE of the basal segment flow. Previous studies have shown that the blood in the LV contains different components during the cardiac cycle; the residual volume refers to the volume that stays at the apex for more than one cardiac cycle, and the KE of the residual volume is small. Corrado’s research also revealed that the volume through the apex was significantly reduced [16].

### Blood flow KE in different segments of the control group

In this study, analysis of the blood flow KE in different segments at the same level in the control group showed no statistically significant differences in the diastolic KE, systolic in-plane KE or diastolic in-plane KE between the anterior and posterior walls of the heart cavity. The difference in blood flow KE between the septal and lateral walls was statistically significant only for diastolic KE and diastolic in-plane KE. Therefore, we preliminarily concluded that the blood flow KE parameters between the anterior and posterior walls were similar. To compare the regional blood flow in the myocardial infarction group, we focused on analysing the anterior and posterior wall blood flow.

LV blood flow has a complex motion and hemodynamic characteristics [17–20]. The statistically significant difference in systolic KE between the anterior and posterior walls in the control group may be related to the presence of an asymmetric vortex in the heart cavity during systole. Goya studied the characteristics of the LV systolic vortex in healthy dogs and reported that the systolic vortex in the posterior basal region had larger vorticity than in the anterior region, this phenomenon can provide reference value for the statistically significant difference in systolic KE between anterior and posterior wall cardiac blood flow in healthy control group [21].

### Regional blood flow KE in the AMI group

The blood flow KE in the infarct segment was lower than that in the noninfarct segment in AMI patients, and the proportion of in-plane KE was increased, which was consistent with Pankaj’s findings [1]. In addition, Arkaet al. [3] reported that patients with adverse ventricular remodelling had increased in-plane KE. The increase in the in-plane KE may be related to pathological blood flow. After MI, LV function was damaged, and the cardiac cavity begins to expand. However, progressive LV impairment and dilatation also cause increased sphericity, which in turn changes flow conditions inside the cavity to a ‘meta-stable’ state with a large, swirling vortex that encompasses the majority of the LV [2]. This vortex flow includes transverse thrusts, which increase the proportion of in-plane KE. In addition, our study revealed that there was no significant difference in diastolic in-plane KE between infarct and noninfarct segments. This finding is consistent with the findings of Arka, which may also be related to changes in diastolic blood flow. Suwa et al. [22] reported that patients with impaired LV function had a greater diastolic vortex than did those with normal LV function.

The purpose of this study is to analyse the changes in KE parameters of regional blood flow in the cardiac cavity of MI patients and to determine whether in-plane KE is highly important in blood flow near the cardiac cavity of the regionally infarcted myocardium. A pathological increase in in-plane KE may exert heterogeneous hemodynamic force on the LV wall, which leads to further expansion of the endocardium and increased endothelial dysfunction [23–26]. This pathological process may be related to the formation of ventricular aneurysms. Ventricular aneurysm [27]is a common complication of AMI. After infarction, necrotic myocardial cells are gradually replaced by fibrous scar tissue, and the infarcted myocardium becomes thinner and bulges outwards, possibly leading to abnormal movement during contraction. To date, there is a lack of research on the relationship between hemodynamic parameters and ventricular aneurysm formation in MI patients. Prospective studies are needed in the future, which may provide hemodynamic insights into the pathophysiology of remodelling after myocardial infarction.

### Study limitations

First, this was a single-centre study with a small sample size. Second, the infarct sites of patients with AMI included in this study were not exactly in the same segment, so the KE between adjacent segments may have an impact. Therefore, the sample size should be expanded for further study to comprehensively evaluate cardiac hemodynamic changes in AMI patients.

## Conclusions

This study provides a methodological reference for the regional analysis of LV flow parameters via 4D Flow MRI. Differences in the KE parameters at different levels and in different segments of the same level in the LV cavity were found in healthy people. In AMI patients, the average KE in the infarct segment decreased, while the proportion of in-plane KE increased. This was a preliminary study analysing regional LV blood flow KE, and further exploration is needed to determine whether regional LV blood flow KE has predictive value for AMI.

## Data Availability

All data generated or analysed during this study are included in this published article.
